# Limitations of Guideline‐Recommended Risk Stratification in Identifying MASLD Patients for Novel Drug Treatments

**DOI:** 10.1111/liv.70281

**Published:** 2025-08-08

**Authors:** Martin Franck, Katharina John, Monika Rau, Andreas Geier, Jan‐Peter Sowa, Jörn M. Schattenberg, Münevver Demir, Heiner Wedemeyer, Klaus Schulze‐Osthoff, Heike Bantel

**Affiliations:** ^1^ Department of Gastroenterology, Hepatology, Infectious Diseases and Endocrinology Hannover Medical School Hannover Germany; ^2^ Division of Hepatology, Department of Internal Medicine II University Hospital Würzburg Würzburg Germany; ^3^ Department of Medicine Universitätsklinikum Knappschaftskrankenhaus Bochum, Ruhr University Bochum Bochum Germany; ^4^ Department of Internal Medicine I University Medical Center Mainz Mainz Germany; ^5^ Department of Internal Medicine II Saarland University Medical Center Homburg Germany; ^6^ Department of Hepatology and Gastroenterology, Campus Virchow Clinic and Campus Charité Mitte Charité University Medicine Berlin Germany; ^7^ Clinic for Gastroenterology and Hepatology University Hospital of Cologne Cologne Germany; ^8^ Department of Molecular Medicine University of Tübingen Tübingen Germany; ^9^ German Cancer Consortium and German Cancer Research Center Heidelberg Germany

**Keywords:** FIB‐4, fibrosis, MASLD, risk stratification, transient elastography

## Abstract

Current guidelines recommend FIB‐4 and transient elastography for non‐invasive risk stratification in metabolic dysfunction‐associated steatotic liver disease (MASLD). These tests can predict the presence or absence of advanced fibrosis, but their accuracy for identifying significant fibrosis in MASLD patients eligible for novel drug treatments remains unclear. In a multicenter cohort of 458 biopsy‐proven MASLD patients, we evaluated the suitability of FIB‐4 for identifying MASLD with histologically significant fibrosis (≥ F2). In a subcohort of 291 patients, we further assessed the diagnostic performance of the guideline‐based sequential use of FIB‐4 and transient elastography. We demonstrate that the recommended risk stratification remains suboptimal for the identification of patients with significant fibrosis, resulting in remarkably high false‐positive and false‐negative rates for FIB‐4 (43% and 26%, respectively). Consequently, guideline‐based risk stratification may misclassify a significant proportion of patients, leading to inappropriate treatment decisions regarding novel MASLD therapies approved for F2/F3 fibrosis.

**Abbreviations**
FDA: Food and Drug Administration
MASLD: metabolic dysfunction‐associated steatotic liver disease
MASH: metabolic dysfunction‐associated steatohepatitis
TE: transient elastography

## 1 Introduction

Metabolic dysfunction‐associated steatotic liver disease (MASLD) is the most prevalent chronic liver disease. It is defined by the presence of liver steatosis and at least one cardiometabolic risk factor [1]. MASLD encompasses a spectrum of conditions, ranging from simple steatosis to metabolic dysfunction‐associated steatohepatitis (MASH). The latter is characterised by histological features of hepatocellular ballooning and lobular inflammation and an increased risk of developing liver fibrosis and cirrhosis [1, 2]. Among these, fibrosis is the main risk factor for both liver‐related and extrahepatic mortality in MASLD [3, 4]. Early diagnosis of fibrosis and appropriate management of MASLD patients are therefore crucial to improving patient outcomes.

Liver biopsy is still considered the gold standard for the diagnosis of MASH and staging of liver fibrosis. Due to its invasive nature, potential complications and high cost, however, it is unsuitable for routine screening or monitoring in clinical settings. Current guidelines therefore recommend a non‐invasive sequential approach for assessing fibrosis in MASLD by using first FIB‐4, an established non‐patented score, followed by transient elastography (TE) or an alternative test, if fibrosis is suspected [1, 2, 5, 6, 7]. The FIB‐4 score is based on routine parameters including age, AST, ALT and platelet count [8] and is easy to apply for fibrosis assessment and risk stratification in MASLD, aiding in disease monitoring and treatment decisions. However, growing evidence indicates a limited accuracy of FIB‐4 in evaluating liver fibrosis, especially in patients with obesity and diabetes [9, 10]. Accurate fibrosis assessment is becoming increasingly important in view of newly developed MASLD drugs, which are currently approved only for patients with non‐cirrhotic MASH and F2/F3 fibrosis. This study therefore aimed to evaluate the diagnostic performance of FIB‐4 and its sequential use with TE in a multicenter cohort of biopsy‐proven MASLD patients to identify patients with a treatment indication.

## 2 Patients and Methods

We investigated 458 patients with biopsy‐proven MASLD (51% male; mean age 47.5 ± 13.5 years), who were not preselected for pharmacotherapy trials, from Hannover Medical School (*n* = 190) and the University Hospitals of Wuerzburg (*n* = 61), Cologne (*n* = 97), Mainz (*n* = 69) and Bochum (*n* = 41). All patients had at least one cardiometabolic risk factor. Histopathological features of MASH including steatosis, hepatocellular ballooning, lobular inflammation and fibrosis stages were assessed by a pathologist [11]. In the entire cohort, the prevalence of fibrosis stages F0, F1, F2, F3 and F4 was 29% (*n* = 134), 34% (*n* = 156), 17% (*n* = 79), 11% (*n* = 51) and 8% (*n* = 38), respectively. According to current guidelines, risk stratification was performed using FIB‐4, which considers a low (< 1.3; for age > 65 years < 2.0) and a high (> 2.67) cut‐off value to rule out or rule in advanced (F3/F4) fibrosis [1, 6, 8].

For intermediate FIB‐4 (1.3–2.67; or 2.0–2.67 for age > 65 years), TE with a cut‐off value of 8 kPa is suggested for further risk stratification [1, 2, 5, 6, 7]. In cases of intermediate FIB‐4, we therefore evaluated the sequential use of FIB‐4 and TE for fibrosis judgement in a sub‐cohort of 291 MASLD patients (52% male, mean age 48.3 ± 13.9 years) with available TE values assessed by FibroScan (Echosens, Paris, France). In this subcohort, patients revealed F0 and F1 in 32% (*n* = 94) and 33% (*n* = 95), F2 and F3 in 16% (*n* = 48) and 10% (*n* = 29), and F4 in 9% (*n* = 25) of cases.

## 3 Results

### 3.1 Histological Fibrosis Stages in MASLD Patients Classified as Low, Intermediate or High Risk by FIB‐4

**FIGURE 1:**
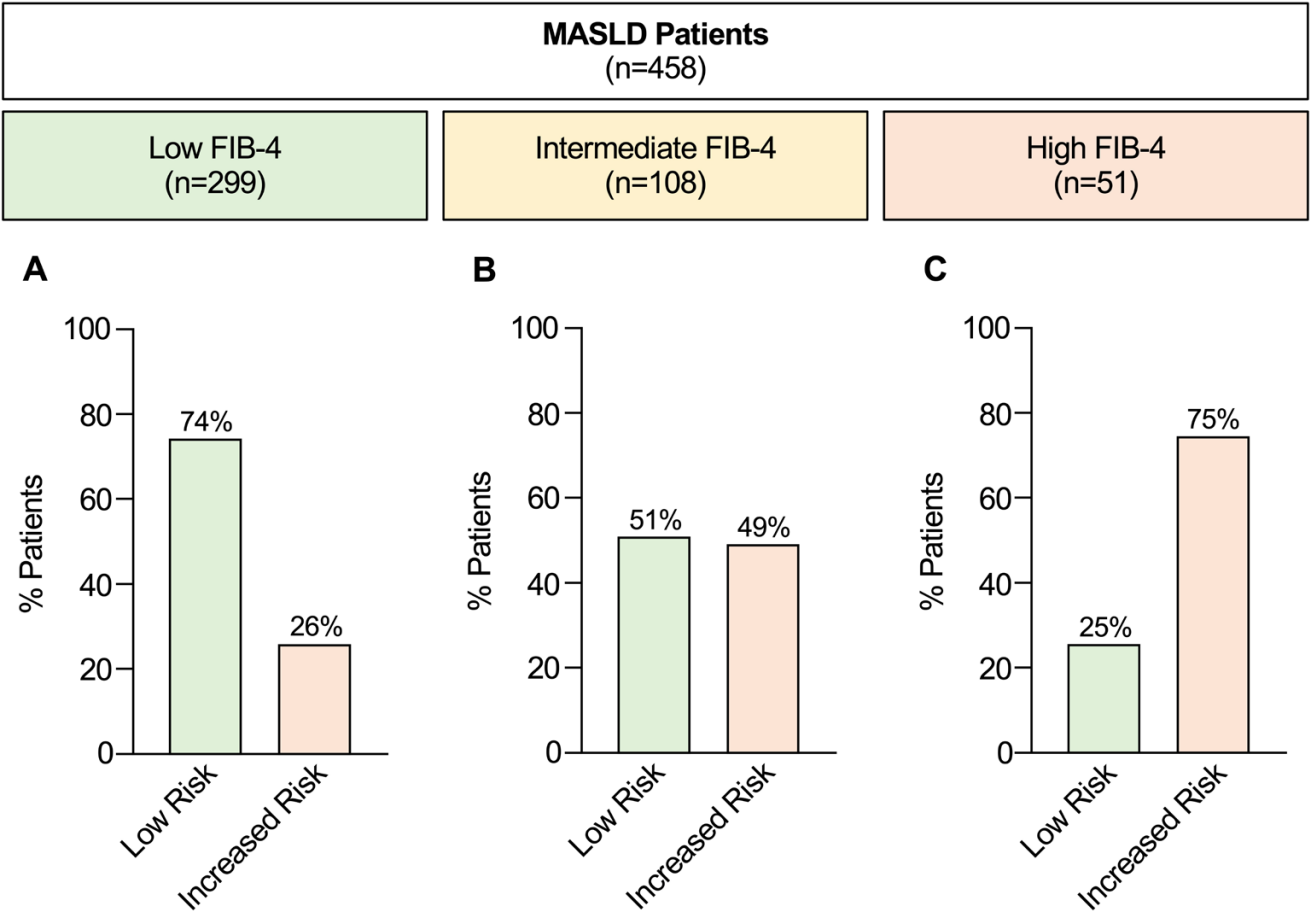
Risk stratification of biopsy‐proven MASLD patients (*n* = 458) by FIB‐4. Patients with a low FIB‐4 (< 1.3, or < 2.0 for age > 65 years) were accurately identified as low risk (histological F0/F1 fibrosis) in 74% of the cases, whereas 26% of patients had an increased risk (histological fibrosis ≥ F2) despite low FIB‐4 (A). Patients with intermediate FIB‐4 (1.3–2.67, or 2.0–2.67 for age > 65 years) were histologically identified as low risk and as increased risk in about half of the cases (51% vs. 49%) (B). Patients with high FIB‐4 (> 2.67) showed an increased risk in 75% of the cases, whereas 25% of patients were not categorised correctly by high FIB‐4 (C).

In the entire multicenter cohort (*n* = 458), 299 patients (65%) revealed a FIB‐4 score < 1.3 (< 2.0 for age > 65 years), indicating a low risk for advanced fibrosis. Among these patients, 74% (222/299) had indeed no (F0) or minimal (F1) fibrosis. However, 26% of patients with a low FIB‐4 were not at low risk and had histologically confirmed significant fibrosis (≥ F2) with fibrosis stages F2 (*n* = 50), F3 (*n* = 19) and F4 (*n* = 8) (Figure 1A). These patients usually remain in primary care for disease monitoring and, due to their low FIB‐4 score, would be overlooked and not considered for further risk stratification.

The intermediate‐risk group (FIB‐4 1.3–2.67, or 2.0–2.67 for age > 65 years) comprised 108 patients (24% of the cohort). Within this group, 51% (*n* = 55) had F0 or F1 fibrosis, while 49% had significant fibrosis: F2 (*n* = 22), F3 (*n* = 19) and F4 (*n* = 12) (Figure 1B). For these patients, guidelines recommend re‐assessment of FIB‐4 within 1 year, or referral to a hepatologist for TE or an alternative test to clarify the fibrosis risk [1, 2, 7].

A total of 51 patients (11%) had a FIB‐4 score > 2.67, indicating high risk. Of these, 75% (38/51) had indeed histologically confirmed significant fibrosis: F2 (*n* = 7), F3 (*n* = 13), and F4 (*n* = 18) (Figure 1C). Patients in this group are typically referred to secondary care for further risk stratification and management. However, 25% of the patients in this group (13/51) lacked histological evidence of significant fibrosis, highlighting the risk of false‐positive cases and inappropriate treatment.

### 3.2 Combination of FIB‐4 and TE for Fibrosis‐Based Risk Stratification

**FIGURE 2:**
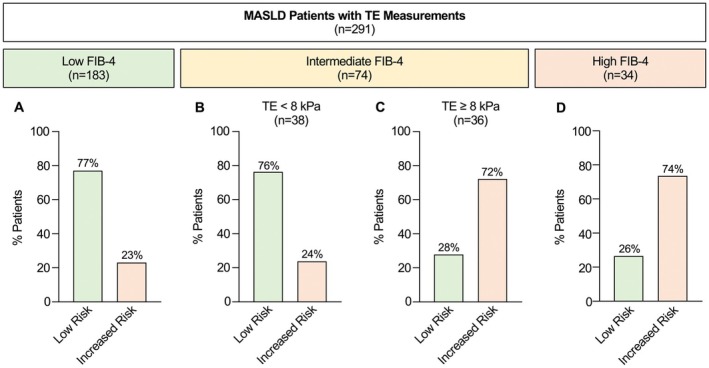
Sequential application of FIB‐4 and transient elastography (TE) for risk stratification of MASLD patients in a sub‐cohort of MASLD patients with available TE values (*n* = 291). Among patients with low FIB‐4 scores (< 1.3, or < 2.0 for age > 65 years), 77% were correctly identified as low risk (histological F0/F1 fibrosis), whereas 23% of the patients had an increased risk (histological fibrosis ≥ F2) despite low FIB‐4 (A). Patients with intermediate FIB‐4 (1.3–2.67, or 2.0–2.67 for age > 65 years) and TE values < 8 kPa or ≥ 8 kPa were correctly classified as low or increased risk in 76% and 72% of cases, respectively, with a false‐positive rate of 28% and a false‐negative rate of 24% (B, C). Among patients with high FIB‐4 score (> 2.67), 74% were accurately identified as having an increased risk, whereas 26% of the patients were misclassified by FIB‐4 (D).

Liver stiffness measurement by TE is recommended for risk stratification in cases of intermediate FIB‐4 [1, 2, 7]. We therefore evaluated the sequential use of FIB‐4 and TE for risk stratification in a sub‐cohort of MASLD patients (*n* = 291) with available TE values. Among patients with low FIB‐4 (*n* = 183), 77% were correctly classified as low‐risk without significant fibrosis (F0/F1). However, 23% exhibited significant fibrosis despite a low FIB‐4 score, with histological stages F2 (*n* = 28), F3 (*n* = 8), and F4 (*n* = 6) (Figure 2A).

From patients with intermediate FIB‐4 (*n* = 74), 53% showed F0/F1 and 47% significant fibrosis (data not shown). Among patients with intermediate FIB‐4 and low TE (< 8 kPa), 76% (29/38) showed no significant fibrosis, but 9 cases were found to have significant fibrosis (F2: *n* = 7, F3: *n* = 2) (Figure 2B). Conversely, in cases with intermediate FIB‐4 and elevated TE (≥ 8 kPa), 72% (26/36) were correctly identified as having significant fibrosis (≥ F2), whereas 28% (10/36) patients revealed no or only mild fibrosis (F0: *n* = 5, F1: *n* = 5) (Figure 2C). Patients with high FIB‐4 (*n* = 34) revealed fibrosis ≥ F2 in 74% (25/34), while 26% (9/34) were overclassified, showing only F0 or F1 fibrosis (Figure 2D). These findings underscore the limitations of non‐invasive risk stratification, revealing notable discrepancies between fibrosis judgement by FIB‐4/TE and histological assessment in a considerable proportion of MASLD patients.

## 4 Discussion

Current guidelines recommend a stepwise, non‐invasive approach for fibrosis risk stratification in MASLD, beginning with the FIB‐4 score and followed by TE in cases of intermediate risk [1, 2, 5, 6, 7]. We evaluated this approach in a biopsy‐proven multicentre MASLD cohort of 458 patients, who were not preselected for pharmacotherapy trials. In this cohort, 74% of patients with low FIB‐4 were correctly classified as low‐risk (F0/F1). However, 26% of patients with low FIB‐4 revealed histologically confirmed significant fibrosis (≥ F2) and thus were misclassified. Similar results were obtained in a sub‐cohort of 291 MASLD patients with available TE values. While previous studies reported a misclassification rate of approximately 10% for patients categorised as low‐risk by FIB‐4, these analyses relied on TE (cut‐off ≥ 8 kPa) rather than liver biopsy as the reference standard for risk assessment [9, 10]. In contrast, our biopsy‐proven study likely offers a more accurate reflection of the misclassification rate by FIB‐4 and/or TE.

Approximately half of our patients with intermediate FIB‐4 revealed significant fibrosis in liver biopsy. In this group, the additional use of TE is recommended to improve diagnostic accuracy. Among patients with intermediate FIB‐4, 76% of those with low TE (< 8 kPa) had no significant fibrosis, while 72% with TE values ≥ 8 kPa had fibrosis stages ≥ F2. However, in cases of intermediate FIB‐4 and low TE values < 8 kPa, more than 20% of patients had significant fibrosis and would be overlooked. *Vice versa*, ~30% of patients with intermediate FIB‐4 and TE values ≥ 8 kPa were overestimated as having significant fibrosis. This finding therefore indicates that the commonly used TE cut‐off value of 8 kPa remains suboptimal for risk stratification of patients with intermediate FIB‐4. We also evaluated the recommended cut‐off value of 12 kPa to rule in advanced fibrosis in MASLD [2]. In the case of intermediate FIB‐4 and TE ≥ 12 kPa, the proportion of patients overestimated for having significant fibrosis was reduced to 22%. However, when using this threshold, 33% of patients with intermediate FIB‐4 and TE values < 12 kPa would be overlooked for having significant fibrosis (data not shown). Combining FIB‐4 with novel blood‐based parameters might improve risk stratification in cases of intermediate FIB‐4 [12, 13, 14, 15]. Nevertheless, further studies are required to validate their clinical suitability and cost‐effectiveness for risk stratification in MASLD.

In our study, high FIB‐4 values were associated with a high false‐positive rate for histologically significant fibrosis, with ~25% of patients in this group having only F0/F1 fibrosis. Similarly, recent studies demonstrated that approximately one third of patients with high FIB‐4 had TE values < 8 kPa, suggesting false‐positive results [9, 16]. In the case of high FIB‐4, further risk assessment is therefore recommended [1, 2].

Non‐invasive risk stratification of MASLD patients is crucial not only for assessing fibrosis and disease monitoring, but also for identifying patients who may benefit from pharmacotherapy. Recently, the thyroid hormone receptor‐β agonist resmetirom gained accelerated FDA approval for patients with non‐cirrhotic MASH and F2/F3 fibrosis [17, 18]. Notably, the presence of steatohepatitis—regardless of fibrosis stage—does not necessarily determine treatment eligibility. According to current guidelines, a liver biopsy is not required for selecting patients for pharmacotherapy, but can be replaced by non‐invasive tests [1]. MASLD patients with FIB‐4 ≥ 1.3 and/or TE ≥ 8 kPa without evidence for cirrhosis have been therefore suggested for pharmacotherapy [6]. However, treating all MASLD patients with elevated FIB‐4 (159 in our cohort) would lead to an inappropriate treatment in 62% of the cases, including 68 patients with F0/1 fibrosis and 30 with cirrhosis (F4). In the MASLD cohort with available TE values, 62% of the patients with elevated FIB‐4 would receive a therapy not approved for this situation (F0/F1: *n* = 48, F4: *n* = 19). Even when restricting eligibility to those with intermediate FIB‐4 score and TE ≥ 8 kPa, 47% would still be inappropriately treated (F0/F1: *n* = 10; F4: *n* = 7). Conversely, at least 20% of patients with low FIB‐4 would be overlooked despite having an indication for therapy.

Our findings therefore indicate that the current non‐invasive risk‐categorization based on FIB‐4 and TE remains suboptimal. It would result in unnecessary treatment of patients without significant fibrosis, as well as in inappropriate treatment of patients with cirrhosis for whom a benefit is unproven and the risk of adverse drug effects may be higher. Moreover, a non‐neglectable proportion of patients with treatment indication would be overlooked. Given the high prevalence of MASLD, liver biopsy is not a viable alternative. Thus, an unmet clinical need remains for more accurate non‐invasive tools to guide treatment initiation and disease monitoring in MASLD.

## Author Contributions

All authors fulfilled the ICMJE definition of authorship.

## Ethics Statement

The study was performed according to the Declaration of Helsinki and the guidelines of the local ethics committees.

## Conflicts of Interest

A.G. is advisory board or steering committee member of AbbVie, Advanz Pharma, Albireo, Alexion, AstraZeneca, Bayer, Boehringer Ingelheim, Bristol Myers Squibb, Falk, Gilead, Heel, Intercept, Madrigal Pharmaceuticals, Merz, MSD, Novartis, NovoNordisk, Pfizer, Sanofi‐Aventis. J.M.S. declares consultant honorary from Alexion, Astra Zeneca, Boehringer Ingelheim, Gilead Sciences, GSK, Madrigal Pharmaceuticals, Nordic Bioscience, Lilly, MSD, Novartis, NovoNordisk, Pfizer, Roche, Sanofi, Siemens Healthineers, Summit Clinical; he received speaker honorarium from Madrigal Pharmaceuticals. H.B. declares consultant honorary from Advanz Pharma, Echosens, Gilead Sciences, GSK, Intercept and Roche Diagnostics; she received speaker honoraria from Falk Foundation, Gilead Sciences, Advanz Pharma and GSK. M.D. is consultant for Gilead and received speaker honorarium from Gilead and Abbvie; she received research support from Echosens. M.R. is consultant for Advanz Pharma and received speaker honorarium from Falk Foundation and Abbvie. H.W. reports honoraria for speaking or consulting from Abbott, AbbVie, Bristol‐Myers Squibb, Boehringer Ingelheim, Eiger Biopharmaceuticals, Gilead Sciences, Janssen, MSD, Novartis, Roche, Roche Diagnostics, Siemens, and Transgene; he received research support from Abbott, Bristol Myers Squibb, and Gilead Sciences. The remaining authors disclose no conflicts.

## Data Availability

The data of this study are available from the authors upon reasonable request.
